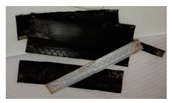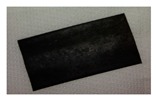# Photo-Oxidative and Soil Burial Degradation of Irrigation Tubes Based on Biodegradable Polymer Blends

**DOI:** 10.3390/polym11091489

**Published:** 2019-09-12

**Authors:** Marco Rapisarda, Francesco Paolo La Mantia, Manuela Ceraulo, Maria Chiara Mistretta, Carmelo Giuffrè, Roberto Pellegrino, Graziella Valenti, Paola Rizzarelli

**Affiliations:** 1 Istituto per i Polimeri, Compositi e Biomateriali, Consiglio Nazionale delle Ricerche, Via Paolo Gaifami 18, 95126 Catania, Italy; marcorapis7@gmail.com (M.R.); valentigraziella1985@gmail.com (G.V.); 2 Department of Engineering, University of Palermo, Viale delle Scienze, Ed.6, 90128 Palermo, Italy; manuela.ceraulo@unipa.it (M.C.); mc.mistretta@gmail.com (M.C.M.); 3 IRRITEC, Cda Scarpitta, 98071 Capo d’Orlando, Italy; carmelo.giuffre@irritec.com (C.G.); roberto.pellegrino@anton-paar.com (R.P.); 4 Anton Paar, Ankerstraße 6, 8054 Graz, Austria

**Keywords:** biodegradable polymers, rheological properties, irrigation pipes, soil burial test, polyesters, Bio-Flex^®^, Mater-Bi^®^, polymer degradation, photo-oxidation

## Abstract

Irrigation tubes based on biodegradable polymers were prepared via an extrusion-drawing process by Irritec and compared to conventional pipes made of high-density polyethylene (HDPE). A commercial polylactide/poly (butyleneadipate-*co*-butyleneterephthalate) (PLA/PBAT) blend (Bio-Flex^®^) and Mater-Bi^®^ were used. The polymers were characterized from rheological and mechanical points of view. Irrigation pipes were subjected to photoaging with continued exposure to UV radiation up to 22 days. The degradability in the soil of irrigation tube samples was studied. The influence of temperature and UV irradiation on soil burial degradation was investigated. A soil burial degradation test was carried out at 30 °C and 50 °C for up to 70 days. The degree of degradation was evaluated from the weight loss percentage. The degradation rate of irrigation tube samples based on Mater-Bi^®^ was higher at 30 °C and was stimulated after 14 days of UV irradiation. Higher temperatures or UV aging encouraged the disintegration in soil of Bio-Flex^®^-based irrigation tubes. Furthermore, tube samples, before and after UV and soil burial degradation, were analyzed by Attenuated Total Reflection-Fourier Transform Infra-Red (ATR-FTIR) spectroscopy.

## 1. Introduction

Nowadays, polymeric materials are widely used in agricultural field for several types of plastic products (i.e., mulch films, containers, filaments, clips, pots, packaging, drip irrigation tubes, greenhouse covers, etc.). Nevertheless, the widespread and increasing use of plastic materials in agriculture and their persistence in the environment contribute seriously to environmental pollution due to plastic wastes. Plastic items manufactured by traditional polymers, such as mulch films, have to be collected after use and recycled or burned for energy recovery. The removal and disposal can be very expensive and difficult to achieve; in addition, plastic residues can persist in the field with a negative environmental impact. Recently, an interesting overview of agricultural plastic waste generation in Europe was published, highlighting this great concern [1].

The worldwide increase in plastic wastes has involved a great deal of strategies aimed at minimizing the negative impact of the cumulative production and consumption of polymeric materials. The reduction of fossil resources and waste accumulation problems have stimulated a growing interest toward replacing conventional plastics by biodegradable ones, including in agriculture. By using biodegradable items, in fact, both recovery and final disposal can be avoided, since they are optimized to degrade in situ.

Biodegradable plastic systems can potentially replace commonly used PE ones in agriculture. Biodegradable items must ensure the same performance of traditional ones, with the advantage of being left in soil or disposed of in industrial composting plants as common organic waste. Indeed, several commercially biodegradable polymers show rheological and mechanical properties suitable for the production of irrigation pipes, as they can be easily processed in extrusion and can also be drawn [2,3,4].

Aliphatic polyesters are among the most employed biodegradable materials in packaging applications as well as in agricultural and sanitary fields [5]. Some studies have been carried out on commercial biodegradable polymers to verify their applicability in mulching or irrigation pipes [6,7]. However, the effect of UV on their performances and degradation rates in soil has not been investigated in depth. In fact, both mulching and irrigation pipes, during their normal use, are exposed to sunlight. Therefore, their photoaging becomes of utmost concern and can affect their performances as well as their biodegradation rates.

The degradation of traditional plastic materials in nature is a very slow process that involves environmental factors and microorganism activities. Polymer degradation implies different processes stimulated by one or more environmental factors, such as heat, light, microorganisms, or chemicals that deteriorate polymers, producing alterations in their properties. The degradation is the result of irreversible structural changes that are usually undesirable or, in some cases, essential, as in biodegradation, or are else induced to support structure determinations, such as in pyrolysis-gas chromatography-mass spectrometry (Py-GC-MS) studies [8,9]. The biodegradation of polymeric materials depends on several chemical-physical features (chemical bonds, composition, crystallinity, morphology, etc.), environmental factors (oxygen, temperature, biological agents, microbial cells, and enzymes), and their combined effect [10].

The degradability in soil of poly(vinyl alcohol) [11], copolyesters [12], and poly(ester amide) [13] film samples has been investigated under controlled soil burial conditions. Despite extensive research on materials, few studies have been focused on polymer degradation due to a combined effect of UV irradiation and soil burial [6,7]. Briassoulis et al. evaluated the degradation behavior of MaterBi^®^-based films and irrigation tubes under real field conditions [7]. However, laboratory tests under controlled conditions can provide more reproducible data for both degradation mechanisms and kinetics.

Biodegradation can be schematically represented in three stages: in the first one, plastic is depolymerized into monomers and oligomers; in the second step, the monomers and oligomers are taken up as biomass; and in the third, the respiration of biomass consumes O_2_ and produces CO_2_ and H_2_O (under aerobic conditions). The measurement of reactant consumption (i.e., the plastic material) does not allow us to prove whether the process has actually been completed or has stopped, for example, at depolymerization. Therefore, all of the standardized methods for determining biodegradation are based on the measurement of respiration, i.e., the conversion into CO_2_ of the carbon initially present in the plastic through the use of the oxidant (O_2_). However, most of the papers in the literature concerning polymer and composite biodegradation have been based on weight loss measurements [10,14,15].

The aim of this work is to report the possible application of two different classes of biodegradable polymers in pipes for irrigation. An evaluation of their performance is reported. Irrigation tubes based on biodegradable polymer blends were prepared by IRRITEC through a proprietary process and compared to conventional pipes made of high-density polyethylene (HDPE). A commercial polylactide/poly(butyleneadipate-*co*-butyleneterephthalate) (PLA/PBAT) blend (Bio-Flex^®^) and a commercial Mater-Bi^®^ blend were used. The polymers were characterized from rheological and mechanical points of view in order to investigate their suitability to be processed in an industrial apparatus to obtain pipes. Irrigation pipes were subjected to photoaging, and their degradability in soil was studied. The influence of temperature and UV irradiation on soil burial degradation was investigated.

## 2. Experiment

### 2.1. Materials

Two biodegradable polymer systems were used in this work: a Mater-Bi^®^ CF04P grade (MB) made by Novamont (Novara, Italy) and a Bio-Flex^®^ F2110 grade (BF) made by FKUR (Willich, Germany). The Mater-Bi^®^ sample had a melt flow rate (MFI) of about 7 g/10 min at 160 °C (under a 5-kg load), and the Bio-Flex^®^ sample (BF) had an MFI of approximately 5 g/10 min (190 °C, 2.16 kg). The composition of the Mater-Bi^®^ biodegradable system was proprietary, and its formulation was based on a combination of biodegradable aliphatic polyesters, biodegradable aliphatic–aromatic copolyesters, and starch [2,7]. The Bio-Flex^®^ was a blend based on PLA and PBAT [16] (ratio ~3:1). A high-density polyethylene (HDPE) sample, utilized for similar applications, was used for comparison. Carbon black (2%) was added in all the compositions. For the soil burial test, tube portions of 2.5 × 5 cm (thickness about 290 µm) were cut. Specimens of 10 × 2.5 cm were used instead for the accelerating weathering test.

### 2.2. Rheological, Mechanical, and Structural Characterization

In order to assess if these two biodegradable polymers could be processed in industrial apparatuses for the production of pipes, a rheological characterization was carried out both in shear and in nonisothermal elongational flow. A rheological characterization was performed in shear flow in a rotational rheometer, a TA (USA) Ares G2, and in a capillary viscometer (a Rheologic 1000 CEAST (Pianezza, TO, Italy)).

The flow curves were measured at *T* = 170 °C for the two biodegradable polymers and at 230 °C for the HDPE.

The behavior of these polymers in nonisothermal elongational flow in this application was very important because the emerging tube from the die extruder was drawn in a molten state to achieve the final desired dimensions. Measures of the properties in nonisothermal elongational flow, of the melt strength (MS), and of the breaking stretching ratio (BSR) were achieved according to procedures described elsewhere [2,3,4].

MS is the force in the molten filament at breaking, while the BSR is the ratio between the drawing speed at breaking and the extrusion velocity in runs in which the drawing velocity increases with steady acceleration. The capillary has a length-to-diameter (*L*/*D*) ratio = 40. The reproducibility of all of the results was good (±5%).

Mechanical properties, the elastic modulus (E), the tensile strength (TS), and the elongation at break (EB) were measured in tensile tests with an Instron (USA) machine at room temperature.

Samples (10 mm/90 mm/~0.6 mm) for mechanical characterization and samples (*D* = 25 mm, thickness about 2 mm) for rheological characterization in shear flow of the unprocessed materials were prepared by compression-molded sheets obtained with a Carver (USA) laboratory press at a temperature of ~170 °C for the two biodegradable polymers and of 230 °C for the HDPE. Attenuated Total Reflection-Fourier Transform Infra-Red (ATR-FTIR) spectra were collected by using a Spectrum Two spectrometer (Perkin-Elmer) using Spectrum software. Spectra were measured through 8 scans at a 4 cm^−1^ resolution.

In order to compare the different effects of photo-oxidation and degradation in soil on the structures, the area ratio between the sums of the peaks at 1550 cm^−1^ and 1850 cm^−1^ and at 2750 cm^−1^ and 3100 cm^−1^, which were related to the stretching of the CH_2_ and CH_3_ groups, was evaluated for the virgin and degraded samples.

### 2.3. Production of the Pipes

The pipes were produced by Irritec (Capo d’Orlando, Italy) through an extrusion-drawing proprietary process. The tube merging from the extruder was drawn to reach the desired dimensions (thickness about 290 μm). The die temperature was ~170 °C for the two biodegradable polymers and 230 °C for the HDPE.

Mater Bi^®^-based pipes were named T1, and the Bio-Flex^®^ ones were called T2.

### 2.4. Accelerated Weathering Test

Specimen portions were exposed to an accelerated weathering test. A QUV Panel system (UVA lamps: λ_max_ = 340 nm) at 60 °C was used (for up to 22 days). Irrigation pipes photo-oxidized at different UV exposure times were selected for a soil burial test in triplicate.

### 2.5. Soil Burial Test

Tests were carried out at 30 and 50 ± 0.1 °C under moisture-controlled conditions. Triplicate specimens at 30 and 50 °C of each irrigation pipe were placed in a series of darkened vessels containing a multilayer substrate [13]. At 30 °C, the specimens of the irrigation pipe (initial weight 0.7 ÷ 1.2 g) were sandwiched between two layers of a mixture of milled perlite (50 g) and commercial soil (200 g) moistened with 100 mL of distilled water (150 mL during the test at 50 °C). The bottom and top layers were filled with 60 g (70 g at 50 °C) of perlite moistened with 120 mL (240 mL at 50 °C) of distilled water. Perlite was added to increase the aeration of the soil and the amount of water retained. A flow of moistened air was supplied from the bottom of each vessel every 12 h for 15 min (every 6 h at 50 °C). The specimens were removed after regular intervals, brushed softly, washed with distilled water several times, and dried under vacuum in the presence of P_2_O_5_ at room temperature to a constant weight [17]. The degree of degradation was evaluated by weight loss (WL) by using the following equation:

WL (%) = (*W*_i_ – *W*_t_)/*W*_i_ × 100, (1)

where *W_i_* is the initial weight of the sample, and *W_t_* is the weight after an established time. Filter paper and polyethylene samples were used, respectively, as a positive and negative control.

## 3. Results and Discussion

### 3.1. Rheological and Mechanical Properties

**Figure 1:**
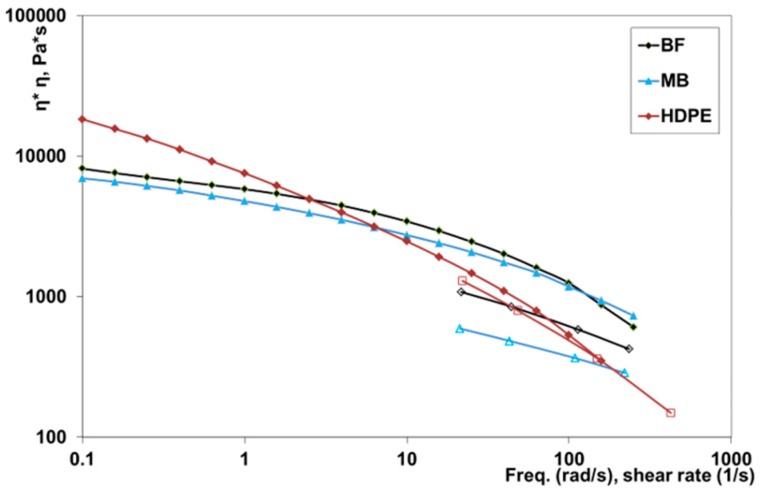
Flow curves of the investigated samples in a rotational rheometer (full symbols) and a capillary viscometer (open symbols).

The flow properties of the two biodegradable samples and of the reference HDPE are reported in Figure 1.

The flow curves obtained in the rotational rheometer and in the capillary viscometer were superimposed, according to the empirical Cox-Merz rule, only for the HDPE sample, while this was not true for the two biodegradable polymers. This phenomenon has already been reported in the literature for similar systems and was in agreement with the results already found in other studies on heterogeneous multiphases [2,18].

The flow curves, moreover, showed that the biodegradable samples present at these temperatures had a viscosity slightly larger and a non-Newtonian behavior less pronounced than the conventional HDPE in the shear rate range (about 100 s^−1^) typical of the extrusion process. However, this difference can be considered acceptable for good extrusion in the production of pipes.

**Figure 2:**
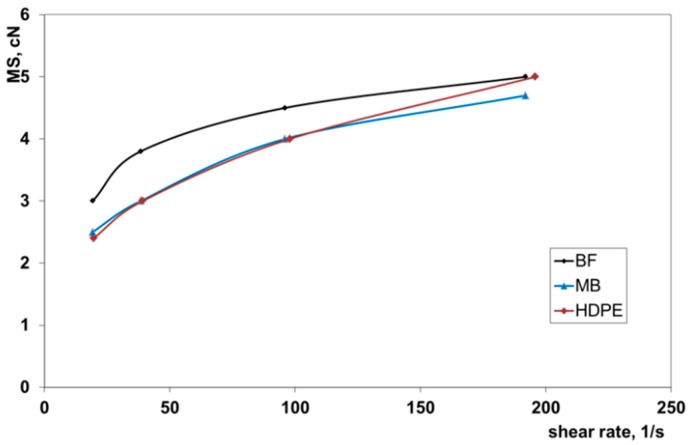
Melt strength (MS) of the investigated samples.

**Figure 3:**
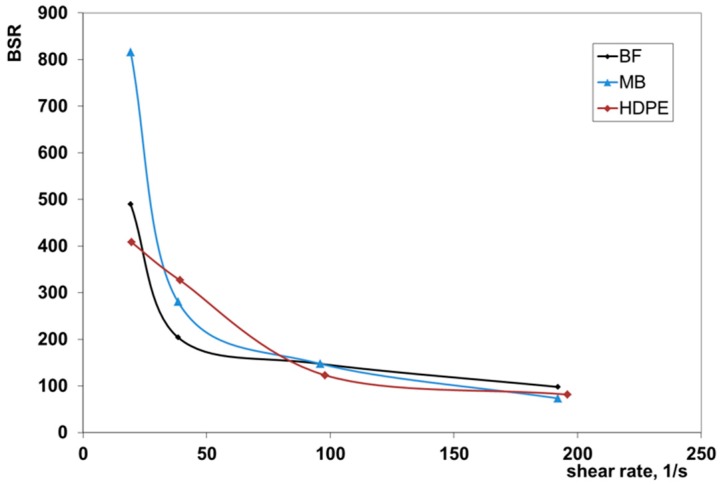
Breaking stretching ratio (BSR) of the investigated samples.

The melt strength (MS) and the breaking stretching ratio (BSR) of the same polymers are reported in the Figure 2 and Figure 3 as a function of the apparent shear rate. In the shear rate range typical of the extrusion process, the melt strength of the biodegradable polymers was very similar to that of HDPE. This means that it is possible to draw the melt to obtain films or pipes that are relatively thick.

High values of the BSR indicated that the molten polymer can be drawn in nonisothermal elongational flow to produce thin films or tubes. All three polymers showed very similar values of the BSR in the shear rate region of the extrusion. This means that the two investigated biodegradable samples will show rheological behavior in the extrusion-drawing process that is very similar.

Mechanical properties, the elastic modulus (E), the tensile strength (TS), and the elongation at break (EB) of the isotropic samples are reported in Table 1.

The elastic modulus of HDPE was higher than that of the two biodegradable samples, and the MB sample showed the lowest value. In addition, the tensile strength and the elongation at break of the HDPE were higher than the biodegradable samples. However, while the TS values of the two biodegradable samples were very similar, the EB value of the Mater-Bi^®^ was much larger than that of the Bio-Flex^®^.

### 3.2. Soil Burial Test

Soil burial degradation tests were carried out under moisture-controlled conditions at 30 ± 0.1 °C and 50 ± 0.1 °C to evaluate the influence of temperature. Triplicate specimens of each irrigation tube were placed in a series of darkened vessels containing a multilayer substrate [13,17]. The polymer films were sandwiched between two layers of a mixture of milled perlite and commercial soil, which were moistened with distilled water. The bottom and top layers were filled with perlite moistened with distilled water. Perlite was added to increase the amount of water retained and the aeration in the soil. A flow of moistened air was supplied from the bottom of each vessel every 24 h for 15 min.

**Figure 4:**
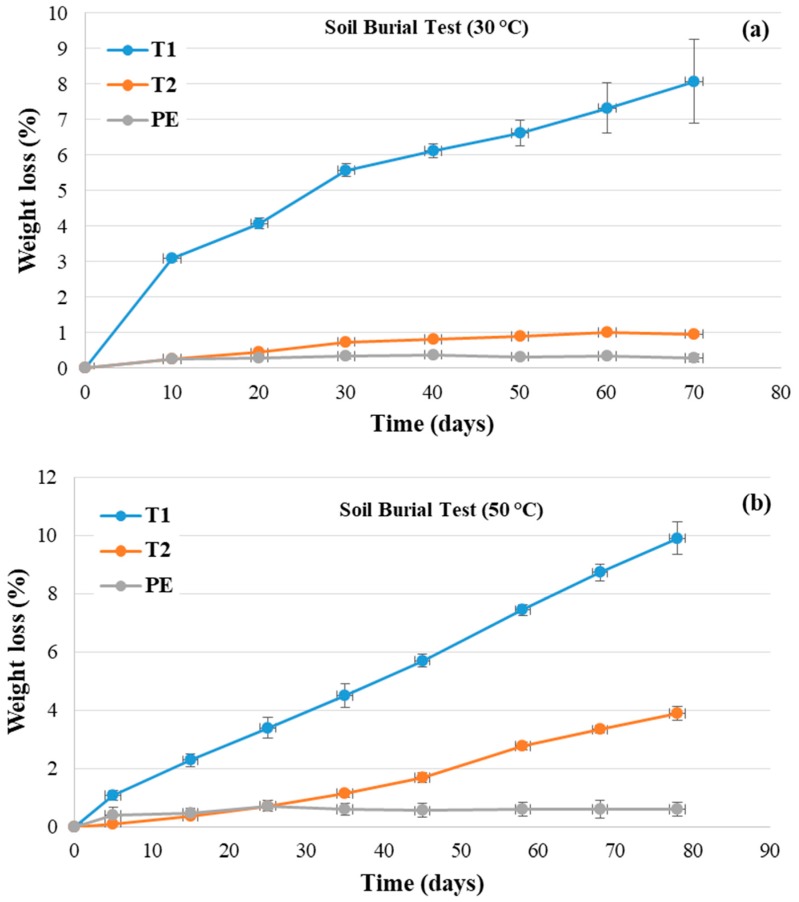
Weight loss (%) versus degradation time for the samples T1, T2, and HDPE at (**a**) 30 °C and (**b**) 50 °C. T1 = Mater Bi^®^-based pipes, and T2 = Bio-Flex^®^-based pipes.

In Figure 4a,b, the average values of the weight loss percentages as a function of degradation time for the samples T1, T2, and HDPE at temperatures of (a) 30 and (b) 50 °C are compared. The carbon black contribution was evaluated and subtracted. T1 (Mater-Bi^®^-based tubes) showed the highest weight losses both at 30 and at 50 °C, with an almost linear trend at 50 °C. On the contrary, for the T2 specimens (Bio-Flex^®^-based tubes), the WL values were remarkably higher at 50 than at 30 °C, reaching 4% at 80 days. By increasing the degradation, the standard deviation grew due to the embrittlement of the samples and the increasing difficulties in the removal of soil from the surface. Unsurprisingly, the WL values for the HDPE specimens were close to zero and remained almost constant throughout the test period. Overall (in agreement with the literature [6]), the higher temperature encouraged the degradation of both biodegradable polymer-based tubes.

Table 2 shows some representative photos of the samples after different soil burial degradation intervals and temperatures. Despite the T1 samples showing the highest WL values, macroscopic degradative effects, such as embrittlement, were observed at 30 °C only after 70 days (Table 2). T2 samples appeared to be stable for a longer time. In the T1 samples, degradation appeared to be increasingly evident after 58 days at 50 °C. At 50 °C, the degradation of the T1 samples produced embrittlement and longitudinal cracks after 68 days (Table 2).

T1 and T2 samples photo-oxidized for 8 and 14 days were selected for the soil burial test at 30 °C. Their weight losses (%) were evaluated for up to 70 days.

**Figure 5:**
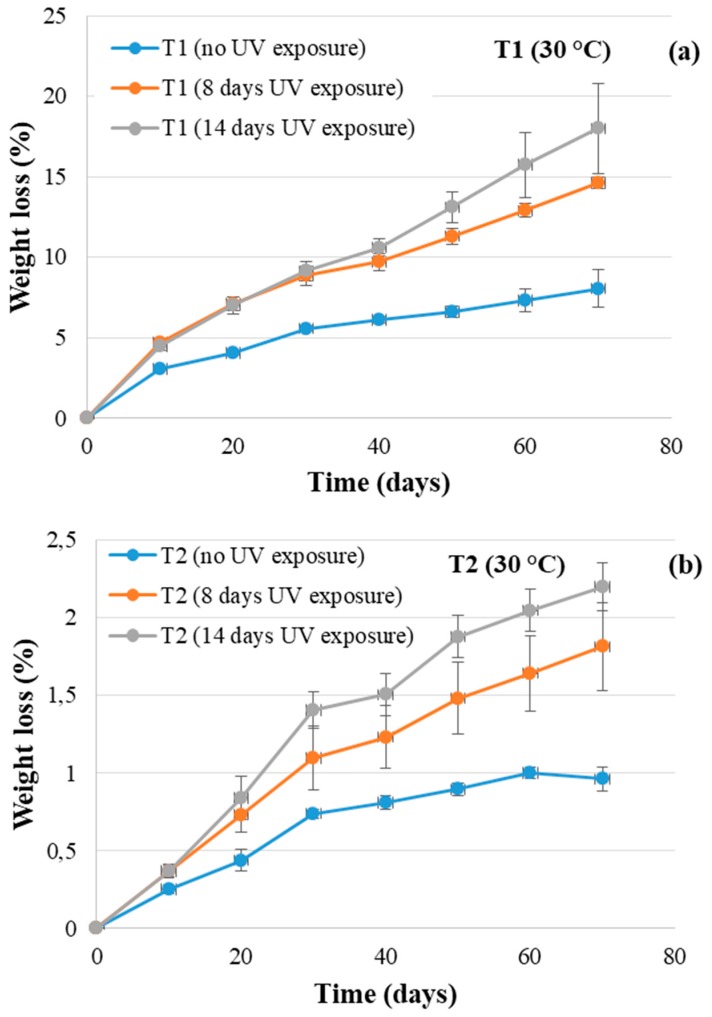
Weight loss (%) versus degradation time for the UV-treated and untreated (**a**) T1 and (**b**) T2 samples. T1 = Mater Bi^®^-based pipes, and T2 = Bio-Flex^®^-based pipes.

In Figure 5, the average values of the WL values were plotted as a function of degradation time at 30 °C for the samples (a) T1 and (b) T2, which were photo-oxidized for 8 and 14 days. For all the samples, photodegradation favored successive degradation in the soil (but more significantly for the specimens of T1) after 30 days of soil burial degradation. Additionally, the WL values increased with the time of UV exposure. Reasonably, UV exposure yielded a molecular weight (MW) decrease producing oligomeric chains, which are more susceptible to the attacks of microorganisms in the soil. In fact, it must be kept in mind that biodegradation proceeds in three stages. Whenever WL is used to monitor the degradation of polymer samples, just the first step is involved, i.e., macromolecular chain depolymerization into monomers and oligomers that are eroded from the surface. UV irradiation enhances and accelerates the formation of monomers and oligomers, increasing consequently the weight loss rate.

The weight loss values that were lower for the T2 samples (i.e., the Bio-Flex^®^-based tubes) than for the T1 specimens (Figure 4b and Figure 5b) could have been related to the presence of PLA, whose biodegradation is MW-dependent. It could be assumed that the higher temperature encouraged the hydrolysis of ester bonds and that UV exposure induced bond-breaking within the macromolecular chains. Consequently, the molecular weight reduction assisted the degradation process in the soil. After the time needed to decrease the molecular weight, the oligomeric products were more easily eroded from the surface of the tubes, and the weight loss values increased. On the other hand, the WL trend at 50 °C, which was close to the *T*_g_ value of PLA, could have affected the weight loss data due to the higher macromolecular chain mobility. The trend of weight loss % versus time with increasing UV exposure (Figure 5) could suggest that chain scission overrules crosslinking reactions. In fact, according to the literature, photo-oxidation gives rise to the rearrangement of polymeric chains, leading, in the case of aliphatic-aromatic copolyesters, to polymeric chain crosslinking and the formation of an insoluble polymeric gel fraction. On the contrary, PLA photochemical reactions produce chain scissions instead of crosslinking [19,20].

**Figure 6:**
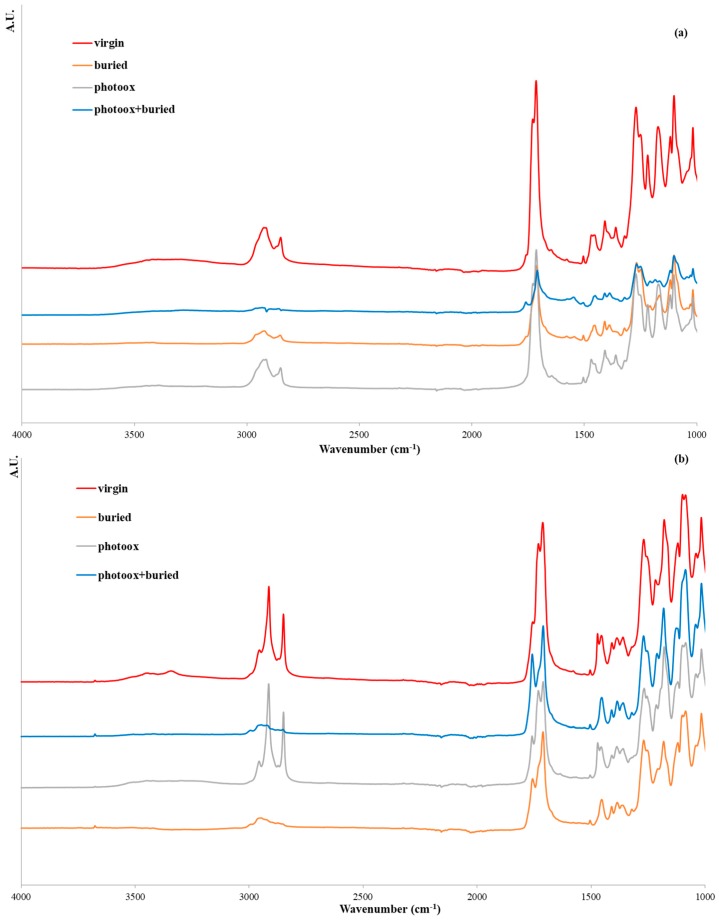
Attenuated Total Reflection-FTIR spectra of virgin, photo-oxidized, soil-buried, and photo-oxidized + buried (**a**) T1 and (**b**) T2 samples. T1 = Mater Bi^®^-based pipes, and T2 = Bio-Flex^®^-based pipes.

Overall (and in agreement with the literature [6]), the photo-oxidation accelerated the soil burial degradation. Therefore, in order to investigate the structural changes induced by the different types of degradation, ATR-FTIR was carried out on virgin, photo-oxidized, soil-buried, and photo-oxidized + buried samples (see spectra reported in Figure 6a,b for T1 and T2, respectively). Unsurprisingly, the spectra of the two samples showed that both photo-oxidation and degradation in the soil increased the ratio of the area in the 1550–1850 cm^−1^ region (of carbonyl groups), as well as the area of the functional groups at 2750 cm^−1^ and 3100 cm^−1^, which were related to the stretching of the CH_2_ and CH_3_ groups. It has to be underlined that carbon back is a UV absorber, and its photostabilizing action is due to the absorption of ultraviolet energy that then is no longer available for the formation of radicals and for the consequent propagation of oxidation reactions [21]. This means that carbon black does not interfere with the photo-oxidative mechanisms of different matrices, and its effect is just to shift photo-oxidation toward longer times, increasing the induction time of the process. Carbon black slows down molecular weight reduction and consequently the degradation process in soil. The preliminary experimental data on the T2 samples without carbon black (data not shown) confirmed our conclusions. The shift toward longer times to start photo-oxidation is relevant from a commercial point of view above all in biodegradable polymeric systems whose performances could be impaired by photo-oxidation processes more than traditional ones would be [6].

**Figure 7:**
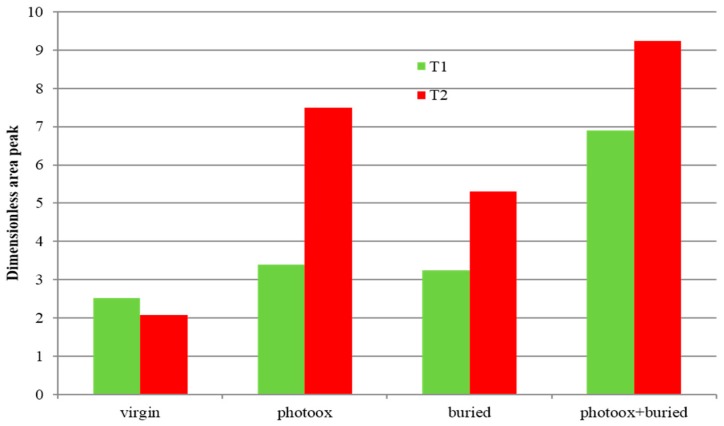
Dimensionless values of area ratio in the 1550–1850 cm^−1^ region for the two biodegradable samples. T1 = Mater Bi^®^-based pipes, and T2 = Bio-Flex^®^-based pipes.

To better follow this trend, in Figure 7 the dimensionless values of the area ratio are reported for the two samples.

According to these data (Figure 7), the Mater-Bi^®^-based sample (T1) showed similar behavior with both photo-oxidation (14 days) and soil degradation (70 days). A synergistic effect of photo-oxidation and soil degradation clearly occurred for both samples. Indeed, after photo-oxidation, the pipe samples seemed much more prone to soil degradation (Figure 5). Interestingly, the Bio-Flex^®^-based sample (T2) showed an apparently peculiar behavior. In fact, the CO increase was more evident in the T2 sample whatever the degradation process, which could have been related to the presence of the PLA component [22]. The increase of the carbonyl groups was an indicator of the photo-oxidation progress as well as of hydrolytic reactions occurring during soil degradation. As a result, T2 was clearly more susceptible to photo-oxidation than to hydrolysis. Even though the CO functional groups increased, due to the combined photo-oxidation and soil degradation, there were more in the T2 sample than in the T1 sample, and the UV irradiation did not accelerate the weight loss rate as it did in the T1 sample (Figure 5). These results were in agreement with the biodegradation process and with our choice of monitoring soil degradation through weight loss. Actually, the T1 sample was much more prone than the T2 sample, since monomers and low-molecular-weight oligomers were originated and removed in the erosion step faster than in the T2 sample.

## 4. Conclusions

The biodegradable polymer systems investigated in this work showed rheological properties both in shear flow and in nonisothermal elongational flow similar to those of a typical polymer used in the production of pipes for irrigation.

As for the mechanical properties, the high-density polyethylene sample showed higher values of elastic modules and of tensile strength and elongation at break than did the two biodegradable samples. However, the values of these mechanical properties were compatible with those required by this application.

Soil burial tests showed that the Mater-Bi^®^-based irrigation tubes were more susceptible to degradation in the soil than the Bio-Flex^®^-based ones were. The increase of the temperature in the soil burial tests had a negligible effect on the Mater-Bi^®^-based tube degradation rate. For the Bio-Flex^®^-based samples, the degradation in soil occurred faster at 50 °C rather than at 30 °C. Additionally, for all of the samples, soil degradation appeared to be encouraged by UV exposure. By comparing the changes in the CO groups measured by the ATR-FTIR spectra to the data of the soil degradation, a larger increase of the CO groups was highlighted for the Bio-Flex^®^-based samples, which showed a minor soil degradation kinetic. The combined results of the soil degradation tests and the ATR-FTIR spectra suggest that the carbon atoms of both materials were oxidized but that the kinetics of soil degradation were faster in the T1 pipe samples than in the T2 pipe samples, whose formation and removal of oligomers containing carbonyl groups was slower.

## Figures and Tables

**Table 1:** Mechanical properties: elastic modulus (E), tensile strength (TS), and elongation at break (EB) of the isotropic samples.

Materials	Modulus (MPa)	Tensile Strength (MPa)	Elongation at Break (%)
MB	81	12.5	400
BF	167	12.4	167
HDPE	316	21.3	700

**Table 2:** Representative photos of the film samples recovered after a soil burial degradation test at different intervals and temperatures.

Temperature Test	Degradation Time in Soil	Sample	
(°C)	(Days)	T1	T2
/	0	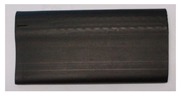	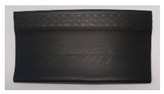
30	60	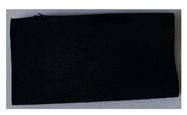	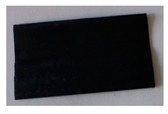
	70	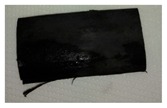	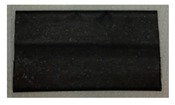
50	58	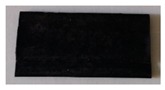	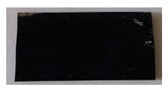
	68	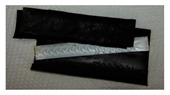	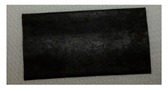
	78